# Structure, light absorption properties and photocatalytic activity of carbon-containing titania nanocomposites synthesized via a facile sol–gel method

**DOI:** 10.1016/j.heliyon.2022.e10199

**Published:** 2022-08-16

**Authors:** Ibrahim Moussa, Hassan Ibrahim, El-Amir M. Emam, Tawfik M. Tawfik

**Affiliations:** a Solid State Physics Department, Physics Research Institute, National Research Centre, 33 El Buhouth St., Dokki, Giza, 12622, Egypt; b Pretreatment and Finishing of Cellulosic Fibers Dept., Textile Research and Technology Institute, National Research Centre, 33 El Bohouth st., Dokki, P.O.12622, Cairo, Egypt; c Faculty of Applied Arts, Textile Printing, Dyeing and Finishing Department, Helwan University, Cairo, Egypt; d Faculty of Applied Arts, Textile Printing, Dyeing and Finishing Department, Benha University, Benha, Egypt

**Keywords:** Sol–gel, Carbon, Titania nanocomposites, Anatase, Rutile oxygen vacancies, Photocatayltic action

## Abstract

Facile and green sol–gel method was used to synthesize carbon-containing titania nanopowder, and diethanolamine (DEA) was used as the in situ carbon source. The titania gel was heat treated at temperatures ranging from 300 to 700 °C. X-ray diffraction (XRD), thermal analysis, and Raman spectroscopy reported no crystalline phase at <325 °C. Crystallization of the anatase phase with traces of brookite phases was observed at T > 325 °C, followed by a transformation to anatase/rutile in the range of 400 °C < T ≤ 650 °C. Finally, the complete phase transformation to the rutile phase occurs at temperatures of T > 650 °C. High-resolution electron microscopy (HREM) micrographs confirm the coexistence of anatase and rutile nanocrystals and amorphous carbon clusters in the composite samples. Chemical element analysis via X-ray photoelectron spectroscopy (XPS) indicated nonstoichiometry in the O/Ti ratio, the presence of (Ti^3+^) oxidation state, and elemental carbon. Thermogravimetric (TG) measurements are the most accurate method to measure the carbon content in samples. UV-vis spectroscopy demonstrated considerable enhancement in the optical absorption properties and electronic structure of prepared samples compared to the pure anatase and rutile. This enhancement is strongly correlated with the structure and composition of prepared samples and consequently depends on the preparation method as well as conditions. Innovative features such as self-cleaning action was demonstrated in carbon containing titanate nanocomposite.

## 1 Introduction

The naturally occurring TiO_2_ contains four polymorphs: anatase, brookite, rutile, and bronze TiO_2_ (B). Although all of these polymorphs comprise TiO_6_ octahedra, their edge and corner-sharing are different from each other. The tetragonal crystal structures of the anatase and rutile polymorphs have space groups I41/amd and P42/mnm, respectively. However, brookite and bronze polymorphs have orthorhombic and monoclinic crystal systems with Pbca and C2/m space groups, respectively [1]. Anatase and rutile TiO_2_ are the most thermodynamically stable phases in the TiO_2_ nanostructure. However, depending on the size and morphology of the nanostructured TiO_2_, the phase transition can prolong to temperatures as high as 1200 °C. In general, the irreversible phase transformation shift from anatase to rutile happens when heated at a temperature of >600 °C in air [2].

TiO_2_ is used in multiple technological applications because of its advantageous properties such as abandonees, low cost, non-toxicity, high chemical and thermal stability, and excellent optical/electronic properties, including paintings and coatings; wastewater treatment; water splitting; hydrogen production; batteries; solar energy harvesting materials; and electronics-, memristors-, and biomedicals-related applications [3, 4, 5, 6, 7, 8]. TiO_2_'s significant bandgap (3 eV), which only permits the material to exploit the absorption of UV light (4 %–5 % of the solar spectrum energy), limits some viable uses. This results in low solar energy utilisation efficiency and a high rate of electron-hole recombination [9].

Numerous methods, including as doping, have been devised to increase the TiO_2_ efficiency when exposed to visible light. with different metal and nonmetal elements [10, 11, 12, 13, 14, 15], semiconductor coupling [16, 17, 18], and surface modifications by which oxygen vacancies and self-doped (Ti^3+^) are incorporated on the surface of nanostructured TiO_2_ [19]. The TiO_2_ surface can be modified by different methods such as wet-chemical reduction methods, including hydrothermal and microwave-assisted hydrothermal methods [20, 21], annealing in a vacuum or an oxygen-deficient environment [22, 23, 24], and annealing under hydrogen gas [25].

Recently, there has been a lot of interest in the surface modification of nanostructured TiO_2_ by carbon or carbonaceous species. Recently, there has been a lot of interest in the surface modification of nanostructured TiO_2_ by carbon or carbonaceous species. Carbon materials have high conductivity and electron storage capacity. They act as surface sensitizers and can efficiently increase the charge transfer rate [26, 27, 28]. Surface modifying carbon and carbonaceous species are innovative means by which surface disorder states are incorporated to narrow the bandgap of nanostructured TiO_2_, suppress the electron–hole recombination rate, and increase visibility and near-infrared (Vis-NIR) light absorption [3, 9]. To overcome the challenges of a multi-step synthesis for large-scale production of the material, or for using affordable facilities, or safer synthetic conditions in the modification process, the realisation of a truly facile, fast, and green synthetic method for preparing highly active carbon modified TiO_2_ nanocomposites remains a challenge.

This study reports a fast, facile, and green sol–gel method for synthesizing carbon-containing titania nanopowder with enhanced optical absorption properties in solar energy applications.

## 2 Experimental and methods

### 2.1 Materials and sample preparation

Titanium tetraisoppropoxide (TTIP 98% Arcos) and diethanolamine (DEA, 99%) were used as starting materials, and anhydrous ethanol was used as a solvent. Commercial TiO_2_ anatase and rutile (99.8% Aladdin Industrial Corporation) were used as reference materials.

Misr Spinning and Weaving Co., El Mahalla El Kubra, Egypt, supplied the textiles utilised, consisting of viscose, linen, viscose, 100 percent cotton, and cotton/PET 50:50 blends. Aldrich-supplied 3-Glycidyloxy propyl trimethoxy silane (GPTMS). Hydrochloric acid, acetic acid, nitric acid, and isopropanol were used without additional purification because they were of analytical quality.

Note that 7.25 g (25 mmol) of TTIP was mixed with 2.65 g of DEA and 20 ml ethanol to produce solution A, and 9 ml bidistilled water was mixed with 11 ml ethanol to produce solution B. Then, solution B was poured into solution A with vigorous stirring. The final solution was reported to be basic with pH ≈ 10 rather than the commonly used acidic solution in titania gel preparation. The hydrolysis and gelation processes were completed at room temperature after mixing for a few minutes. The molar ratio of TTIP: DEA: H_2_O: ethanol was 1:1:20:20. The gel obtained was dried in a 150 °C oven for 12 h. The dried gel was heat treated in static air for 2 h at different temperatures from 300 °C to 700 °C. Each sample is labeled as TDx where x represents the calcination temperature.

#### 2.1.1 Treatment of cotton fabrics with carbon containing titania nanocomposites

Linen, viscose, 100 % cotton, and cotton/PET fabrics were soaked for 5 min in a finishing solution comprising 200 ml of hydrolyzed 3-Glycidyloxy propyl trimethoxy silane (GPTMS) and 0.25 g of carbon-containing titania nanocomposites. After 20 min, a few drops of N-methylimidazole were added as a catalyst, and the samples were dried at room temperature before being cured at ninety degrees Celsius for 20 min. The samples were then rinsed twice with distilled water and dried at room temperature [29].

#### 2.1.2 Self-cleaning action of carbon containing titania nanocomposites loaded cotton fabrics

By exposing samples containing adsorbed Methylene blue (MB) to sunlight, the self-cleaning effect of the Carbon-containing Titania nanocomposites was determined. One half of each stain was exposed to sunlight for 12–48 h, while the other half was covered with black paper to avoid exposure to sunlight. The exposed portion of the stain was compared to the covered portion in terms of its ability to clear itself. The colour strength, as defined by the K/S value, of untreated and Carbon-containing Titania nanocomposites-loaded fabric samples was determined. K/S values are directly proportional to the dye concentration on the substrate. The decline in K/S values is a direct result of the dye stain degradation [30], according to equation [1]:

(1) $$Extent\;of\;discoloration\left(\%\right)=\frac{\left(K/S\right)a}{\left(K/S\right)b}x100$$

where (K/S)a represents the colour intensity immediately after being exposed to daylight and (K/S)b represents the colour intensity just before being exposed to daylight.

### 2.2 Characterization

XRD patterns were recorded in the range of 20^o^ –80^o^ at a scan rate of 4^o^/min using the Pananalytic X'pert diffractometer with Cu*K*_α_ radiation (*λ* = 1.5406 Å). The Rietveld refinement method was implemented to calculate the microstructure and weight fraction of phases using FullProf [31]. All patterns were simulated with a model including the three TiO_2_ polymorphs. The crystallographic parameters used to conduct the structural refinement for each phase were determined using the crystallographic information files (CIF) of anatase, rutile, and brookite phases [32, 33, 34]. The Tompson–Cox–Hastings pseudo-Voigt function with axial asymmetry is considered to be fitted with the measured data [35]. The refinement is evaluated using the weighted profile and expected R-factors: (R_wp_) and (R_exp)_ [36, 37]. The goodness-of-fit or χ^2^ is the R_wp_/R_exp_ ratio, which should be ∼1.

Equation [2] is used to determine the weight fractions (Wi) of the phases found in the powder:

(2) Wi={SjZjMjVjtj∑iSjZjMjVjtj}

where Mj is the mass of the formula unit, Vj is the volume of the unit cell, Sj is the scaling factor, Zj is the number of formulas per unit cell, and tj is the Brindley particle absorption contrast factor for the phase j [38]. Through the use of the Williamson-Hall (W–H) equation, the crystallite size is determined from equation (3) [39].

(3) $$\frac{\beta *\cos\left(\theta_{hkl}\right)}{\lambda }=\frac{K}{D}+4\varepsilon \frac{\sin\left(\theta_{hkl}\right)}{\lambda }$$

where *θ*_*hkl*_ is the peak position, *β* is the broadening (FWHM), *λ* is the wavelength, *K* is a constant, which is generally considered 0.9, ε is the strain, and the crystallite size *D* is calculated from the intercept of the straight line (W–H plot) with the $\frac{\beta *\cos\left(\theta_{hkl}\right)}{\lambda }$ axis [40].

Thermogravimetric analysis (TGA) and differential scanning calorimetry (DSC) were performed on a NETZSCH SAT449 F5 thermo-gravimetric (TG) analyzer, and measurements were conducted in a temperature range from 40^o^C–1000 °C with a heating rate 10 °C/min in air

Raman spectra were recorded at room temperature from 100 to 1000 cm^−1^, and the samples were excited with a 785-nm-sized laser beam.

HRTEM images of samples were obtained using a Tecnai G^2^ F30 high-resolution transmission electron microscope with an attached EDAX unit.

The surface chemical composition of samples was evaluated using Kratos AXIS.

ULTRA DLD X-ray photoelectron spectroscopy (XPS). XPSPeak41 free was used for photoelectron peaks fitting.

The UV-vis absorbance and diffuse reflectance spectra (DRS) in the range from 200 to 800 nm were recorded using a spectrophotometer.

## 3 Results and discussion

The hydrolysis and condensation of titanium tetra isopropoxide in aqueous media can be used to generate titanium dioxide nanoparticles (TiO_2_NPs) using the sol-gel method.

In the presence of water, alkoxides are hydrolyzed and then polymerized to create a three-dimensional oxide network. The following diagram can be used to represent these reactions.

**Figure:**
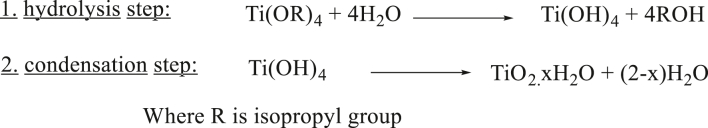

### 3.1 XRD analysis

**Figure 1:**
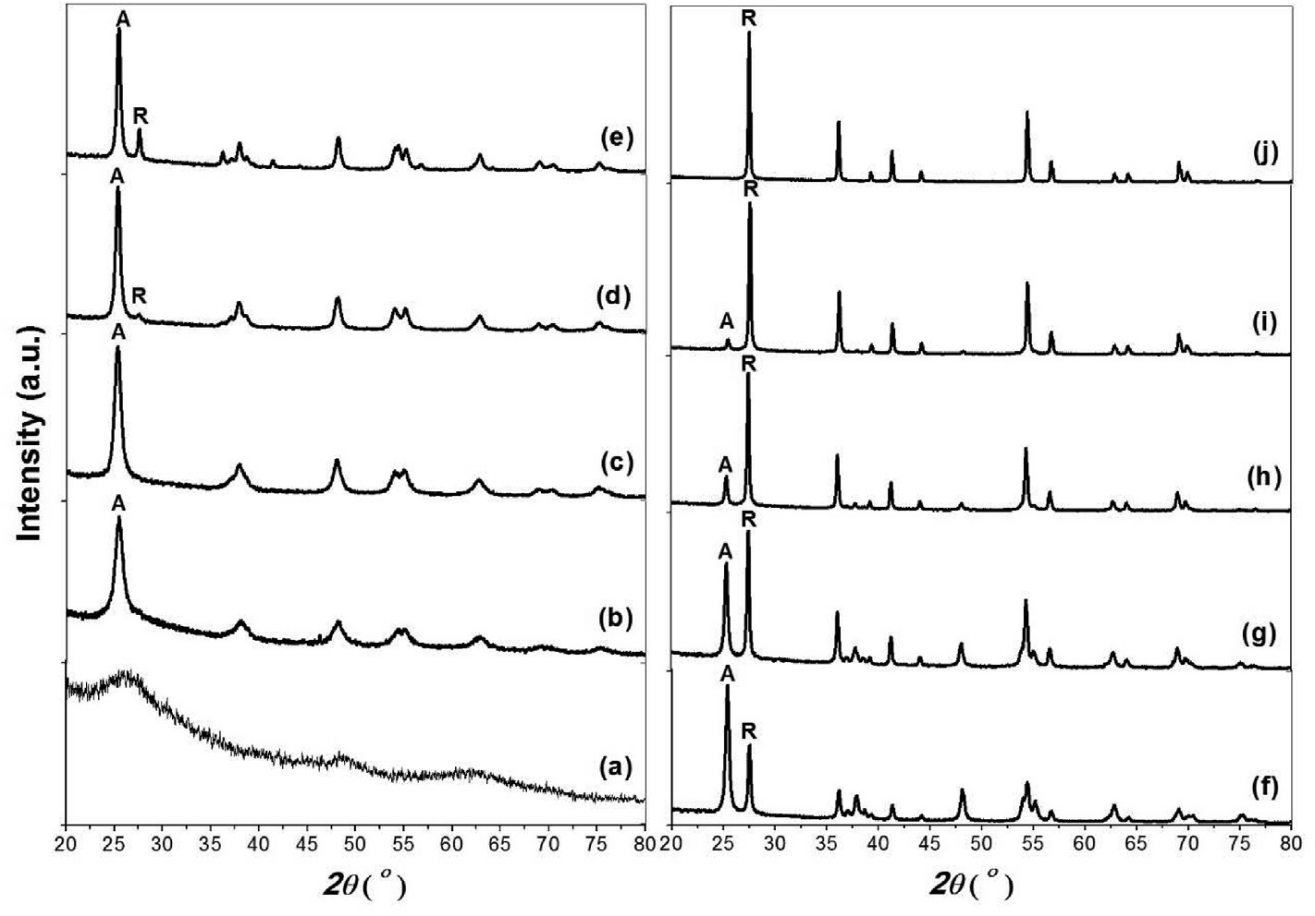
XRD patterns for titania powder heat treated at temperatures from 300 °C to 700 °C; (a) 300 °C, (b) 350 °C, (c) 400 °C, (d) 425 °C, (e) 475 °C, (f) 525 °C, (g) 550 °C, (h) 575 °C, (i) 650 °C and (j) 700 °C.

Figure 1 shows the XRD patterns of certain selected samples heated at different temperatures from 300 °C to 700 °C. The figure shows that the sample TD300 is amorphous, no crystalline phases are detected, while the other samples are reported to be crystalline and have the characteristic peaks of anatase A (101) (JCPDS card no. 00-21-1272) and R (110) of rutile phase (JCPDS card no. 00-21-1276). However, the sample TD700 has been observed as a pure rutile phase. These observations report that the anatase phase is formed at a temperature of >300 °C. The rutile phase is then formed at temperature 400 < T < 425 °C, and the complete transformation from anatase to the rutile phase occurs at a temperature of >650 °C.

**Figure 2:**
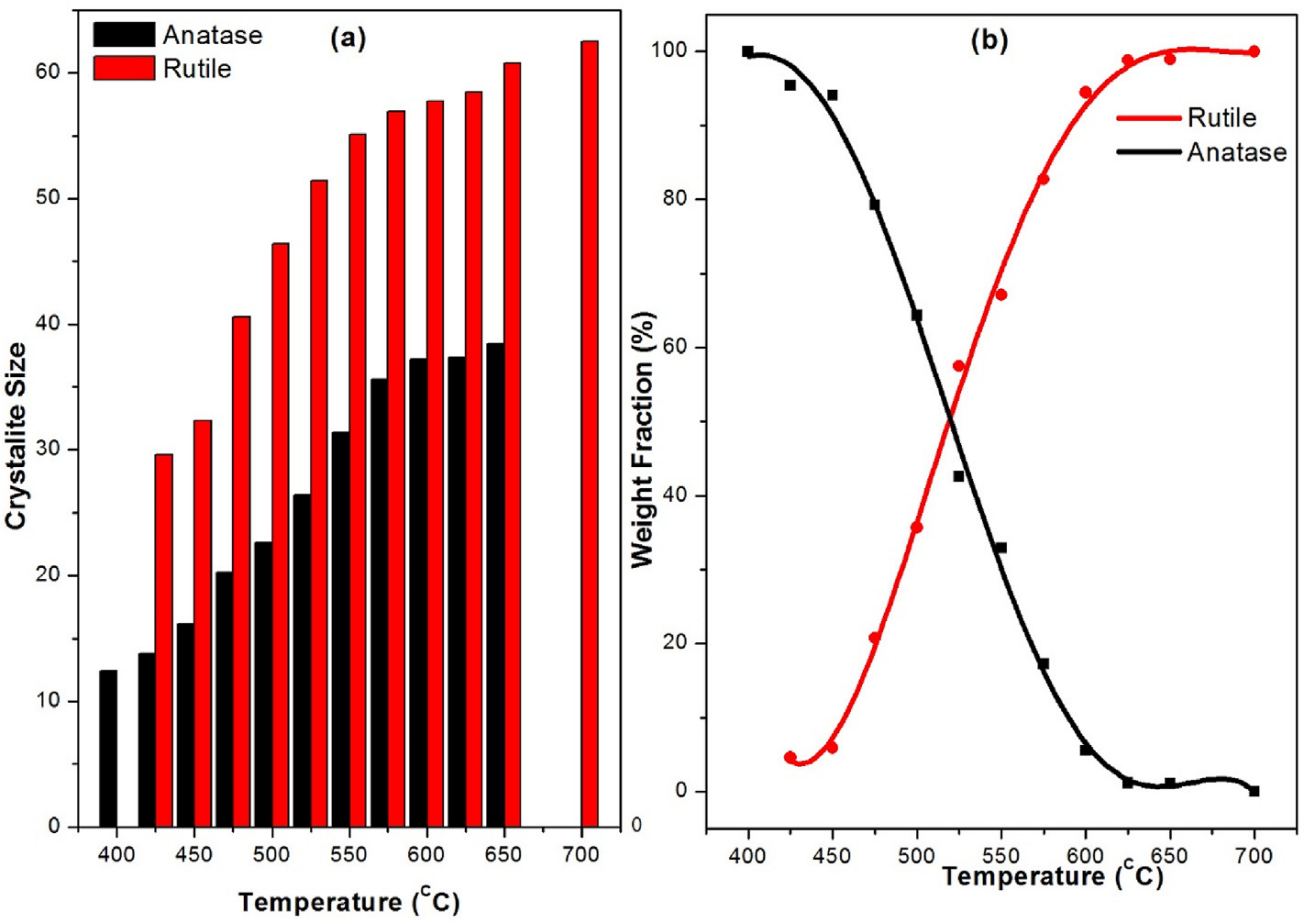
(a) The crystallite sizes of anatase and rutile phases. (b) Weight fraction anatase (black line) and rutile (red line) as functions of temperature.

The acquired Rietveld refinements for all samples heat treated at temperatures from 400 °C to 700 °C agree with the experimentally measured profiles (R_wp_ < 10%, R_exp_ < 9%, and 1 < χ^2^ < 2). Because of refinement, the sample TD400 is reported not to be a pure anatase phase; however, it is a mixture of anatase (98%) and brookite trace (2%) with crystallite sizes of 9.50 and 8.25 nm, respectively. The sample TD700 is a pure rutile phase with a crystallite size of 62.52 nm. All samples heat treated at temperatures from 425 °C to 650 °C are mixtures of anatase and rutile phases. Figure 2 shows the Rietveld refinement of sample TD550 and shows the process for all the described samples.

Table 1 shows the data of refined lattice parameters, the refined weight fraction of phases present, and crystallite size calculated by the W–H plot and Debye–Scherrer's equation.

**Table 1:** Lattice parameters, crystallite sizes by W–H Plot and Debye–Scherrer (D.Sch.), weight fraction by Rietveld refinement (R-method), Suppr-Mayer (S–M) method, and Raman data for anatase and rutile phase of all samples (400 °C–700 °C).

Sample	Anatase							Rutile						
	Lattice Parameters (Å)		Crystallite Size (nm)		Weight Fraction (%)			Lattice Parameters (Å)		Crystallite Size (nm)		Weight Fraction %		
	a	b	W–H Plot	D.Sch. Eq.	R. method	S-M method	Raman	a	b	W–H Plot	D.Sch. Eq.	R. method	S-M method	Raman
TD400	3.7888	9.5022	12.41	10.65	98	0	100	x	x	x	x	x	x	x
TD425	3.7877	9.5023	13.81	12.69	95.42	96.15	100	4.5912	2.9589	29.61	26.39	4.58	3.85	x
TD450	3.7869	9.5093	16.16	14.54	94.12	95.07	100	4.5918	2.9586	32.32	27.24	5.88	4.95	x
TD475	3.7852	9.5110	20.28	18.5	79.24	81.56	75.79	4.5925	2.9588	40.55	29.37	20.76	18.44	24.21
TD500	3.7843	9.5126	22.61	21	64.34	67.12	68	4.5921	2.9583	46.36	30.91	35.66	32.88	32
TD525	3.7836	9.5128	26.39	23.05	42.54	45.79	45.1	4.5929	2.9582	51.41	33.28	57.46	54.21	54.9
TD550	3.7834	9.5183	31.36	24.28	32.9	36.55	32.22	4.5924	2.9583	55.05	36.38	67.1	63.45	67.78
TD575	3.7821	9.5162	35.62	27.07	17.26	19.17	32	4.5928	2.9588	56.91	41.39	82.74	80.83	68
TD600	3.7820	9.5164	37.19	29.85	5.54	6.87	x	4.5929	2.9584	57.75	45.87	94.46	93.13	100
TD625	3.7808	9.4918	37.35	30.52	1.17	1.41	x	4.5927	2.9580	58.44	46.44	98.83	98.59	100
TD650	3.7866	9.4868	38.45	31.41	1.06	1.27	x	4.5927	2.9589	60.8	46.76	98.94	98.73	100
TD700	x	x	x	x	x	x	x	4.5927	2.9584	62.52	47.87	100	100	100

As shown in Table 1, there is no noticeable change in lattice parameters in both phases (anatase and rutile) because of the effect of heat treatment at different temperatures. The crystallite sizes calculated using the W–H plot were larger than those calculated from Scherrer's equation. The reason is that the former method does not consider broadening because of the strain effect. Figure 2(a) shows the crystallite sizes calculated with a W–H plot as a function of temperature, thus showing an increase in crystallite size of both phases with temperature from 12.41 to 38.45 nm for the anatase phase and from 29.61 to 62.52 for the rutile phase.

Figure 2(b) shows the weight fractions of rutile and anatase phases calculated using the Rietveld refinement method as functions of temperature, demonstrating that the desired anatase/rutile ratio in titania nanocomposite can be easily controlled with changing temperature in a lower temperature range.

### 3.2 (TG–DSC) analysis

**Figure 3:**
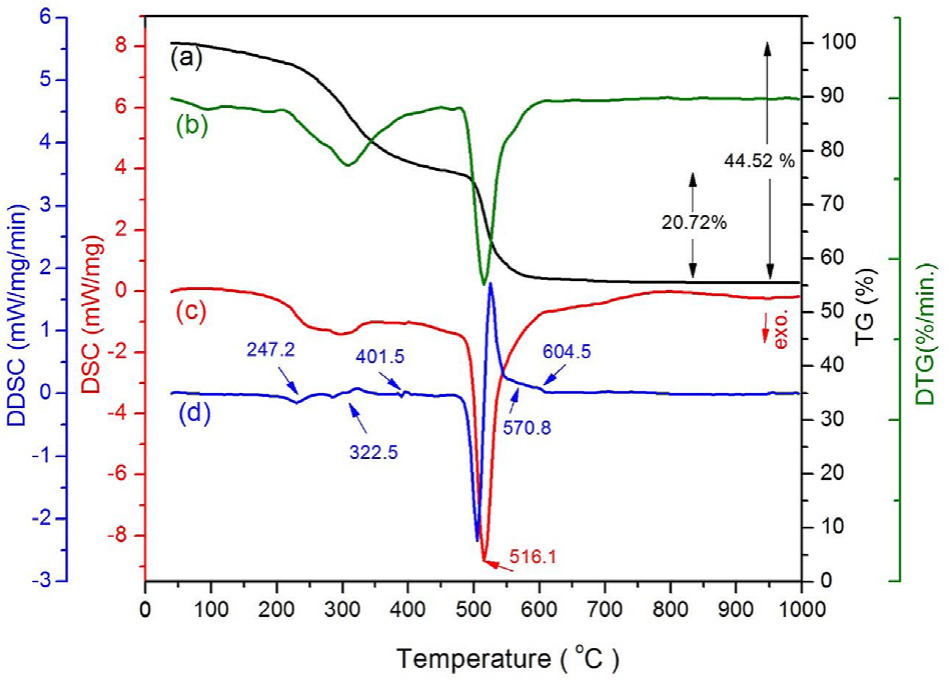
The thermograms from 40 °C to 1000 °C of titania xerogel dried at 150 °C for 12 h; (a) TG black, (b) DTG green, (c) DSC red, and (d) DDSC blue lines.

The thermal behavior of titania gel derived from the TTIP–DEA–H_2_O–ethanol system was examined using simultaneous TG–DSC thermal analysis. Figure 3 shows the resulting TG, DTG, DSC, and DDSC for titania dried gel.

The TG curve shows a total mass loss of 44.52% in two marked steps, the first step with a mass loss of 23.6% in the temperature range from 40 to 454.8 °C, the second step with a mass loss of 20.72% in the range from 454.8 to 1000 °C. The DTG curve represents the mass loss rate, it shows two peaks corresponding to the two mass loss regions in TG curve, the first peak has maximum mass loss rate at 307.9 °C, and the second peak has a maximum rate at 516.1 °C. The DSC curve has one small and broad endothermic peak attributed to evaporation of adsorbed water molecules. Also, it shows a complex exothermic peak with sharp maximum at 516.1 °C, this complex peak resulted from superposition of several exothermic peaks of different thermal processes over a wide range of temperature. To understand this thermal behavior, DDSC curve was derived, it exhibits 6 transition temperatures over the range from 200 to 650 °C, the 1^st^ is at 247.2 °C assigned to decomposition of DEA molecules, the 2^nd^ and 3^rd^ transition points are at 322.5 and 401.5 °C the second point representing the formation of crystalline anatase and brookite, the formation of Brookite phase is attributed to the decomposition of titanium-organic complexes [41]. The third transition point represents transition from unstable brookite to stable rutile phase [42, 43]. The 4^th^ transition temperature at 516.1 °C is assigned to decomposition of titanium-organic residues mainly TTIP-DEA complexes and associated species [44], the 5^th^ transition point at 570.8 °C may represent oxidation of residual carbon structures and release of CO_2_ gas, the last transition point at 604.5 C may represent the removal of oxygen vacancies formed on the surface of due to decarbonization.

Carbon content was estimated by measuring the thermogram (TG) for all samples heat treated from 400 to 700 °C and the weight fraction of carbon (MW%) in titania nano powder as given in Table 2. It is clear that, the carbon content decreases with increasing calcination temperature till vanish at high temperatures T ≥ 700 °C.

**Table 2:** Carbon content (MW%), fractional composition (MW%), O/Ti ratio, and bandgap energy of anatase and rutile phases in C/TiO_2-δ_ nanocomposite samples heat-treated at different temperatures.

Sample	Carbon	Anatase			Rutile		
	Fraction %	Fraction %	O/Ti	Abs. Edge (eV)	Fraction %	O/Ti	Abs. Edge (eV)
TD400	10.7	87.5	1.95	3.16	x	x	x
TD425	6.71	89	1.9	3.15	4.52	1.95	x
TD450	5.96	88.5	1.9	3.11	5.53	1.95	x
TD475	5.21	75.11	1.9	3.07	19.68	1.9	2.93
TD500	4.66	64.34	1.85	3.05	35.66	1.85	2.9
TD525	4.05	40.82	1.75	2.82	55.13	1.8	2.7
TD550	3.3	31.81	1.8	2.9	64.89	1.85	2.87
TD575	2.35	16.85	1.85	2.9	80.8	1.9	2.9
TD600	1.83	5.44	1.93	x	92.73	1.93	2.93
TD625	1.45	1.15	1.95	x	97.4	1.95	2.94
TD650	1.11	1.05	2	x	97.85	1.95	2.94
TD700	x	x	x	x	100	2	3

### 3.3 Raman spectroscopy

All three TiO_2_ crystal structures have the same fundamental structural unit [TiO_6_] octahedron but with different arrangements and links, consequently having different characteristic Raman modes. Anatase has six (1A_1g_ + 2B_1g_ + 3E_g_) Raman-active modes, while rutile has four (A_1g_ + B_1g_ + B_2g_ + E_g_) modes [45]. However, Brookite has predicted 36 (9A_1g_ + 9B_1g_ + 9B_2g_ + 9B_3g_) Raman-active modes [46]. The E_g_ mode is assigned to the symmetrical stretching vibration of O–Ti–O bonds in TiO_2_, B1g mode corresponds to a symmetrical bending vibration of O–Ti–O, and A_1g_ mode refers to the anti-symmetric bending of O–Ti–O bonds [47].

**Figure 4:**
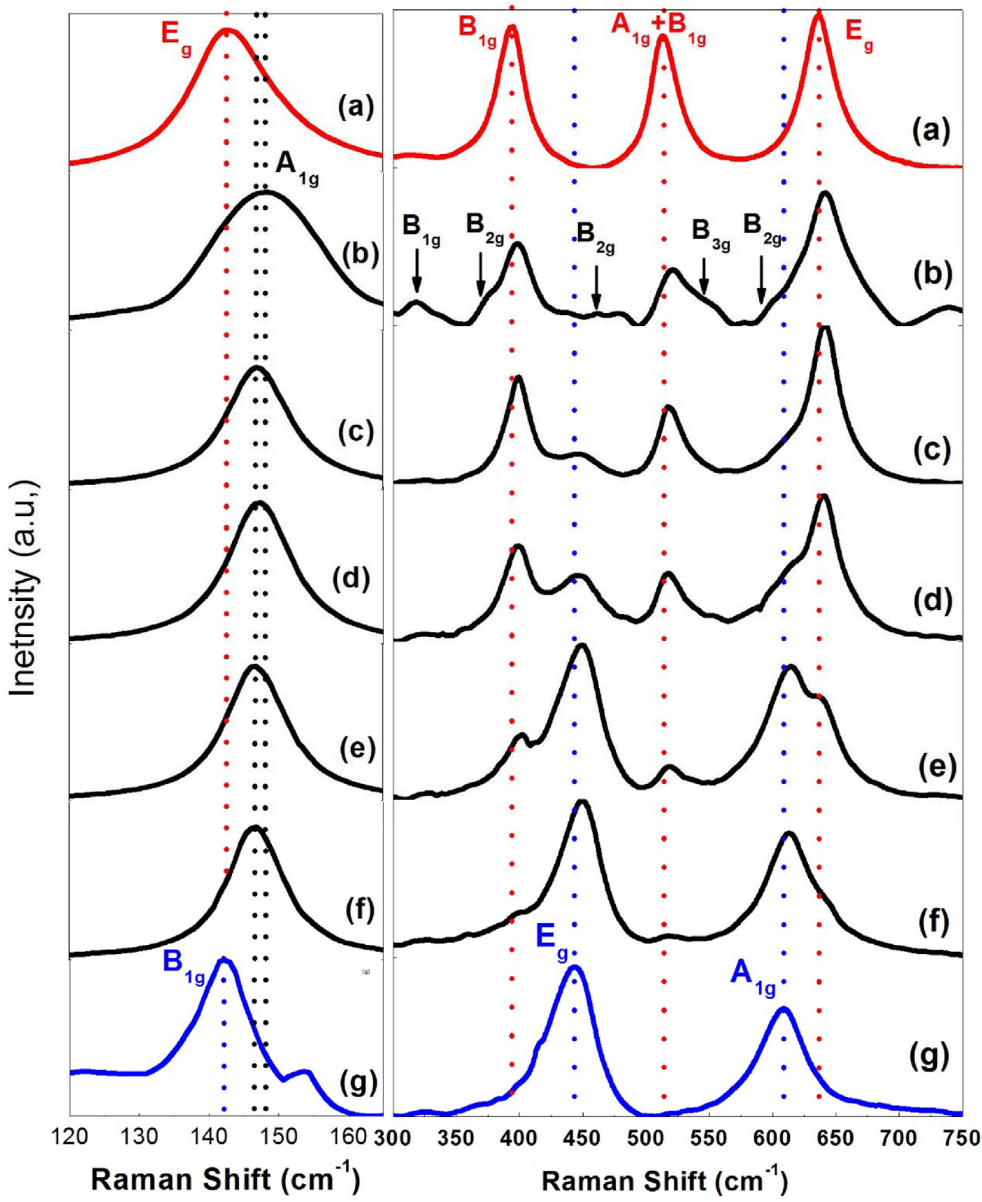
Characteristic Raman-active modes for commercial anatase (a), carbon containing composite samples; (b) TD400, (c) TD 475, (d) TD500, (e) TD550, (f) TD600, and commercial rutile (g).

Figure 4 shows the Raman spectra of certain selected titania composite samples and pure commercial anatase and rutile powder ranging from 100 to 200 cm^−1^ and from 300 to 750 cm^−1^.

As shown in Figure 4, curve (a) shows a strong sharp peak representing the characteristic Raman active mode of the pure anatase phase at 143.1 cm^−1^ with FWHM = 14.5 cm^−1^, and three peaks positioned at 393.3 cm^−1^ (B_1g_), 513.4 cm^−1^ which is the result of two components A_1g_ and B_1g_ of the anatase phase [48], and 635.3 cm^−1^ (E_g_). Curve (g) shows the weak B_1g_ and the characteristic modes E_g_ and A_1g_ modes of the pure rutile phase at 142.3, 442.8, and 608.3 cm^−1^, respectively. Raman spectra of carbon-containing titania samples; curves from (b to f) show movement in a position toward higher energy (blue shift) and change in peak broadening, particularly the characteristic peak of the E_g_ mode compared to the pure anatase phase. Curve (b) represents the Raman spectrum of sample TD400 fired at 400 °C. The characteristic anatase E_g_ is convoluted with the strong characteristic A_1g_ (153 cm^−1^) of the brookite phase (confirmed with XRD) [39], thus resulting in a peak located at 148.3 cm^−1^ with FWHM equals 18.5 cm^−1^. Some other brookite Raman active modes such as B_1g_ (320 cm^−1^), B_2g_ (366, 463, 584 cm^−1^), and B_3g_ (545 cm^−1^) appear. In curves from c to f, the anatase E_g_ (143.1 cm^−1^) is convoluted with the Rutile B1g (142.3 cm^−1^), and the resulting peak is blue-shifted to higher energy at 146.8 cm^−1^. The blue shifts of Raman bands in C/TiO_2-δ_ nanocomposites could be attributed to stress caused by probably nonstoichiometric oxygen deficiency [49] and/or lattice mismatch between different phases present in composites such as carbon/brookite/anatase in the sample TD400 represented by curve b, and carbon/rutile/anatase in samples represented by curves from (c to f). The FWHM for each sample was less than that of pure anatase, and it declines as the calcination temperature rises. The change in peak broadening is attributed to the size effect where broadening is inversely proportional to the crystallite size [50].

### 3.4 High-resolution transmission electron microscopy

**Figure 5:**
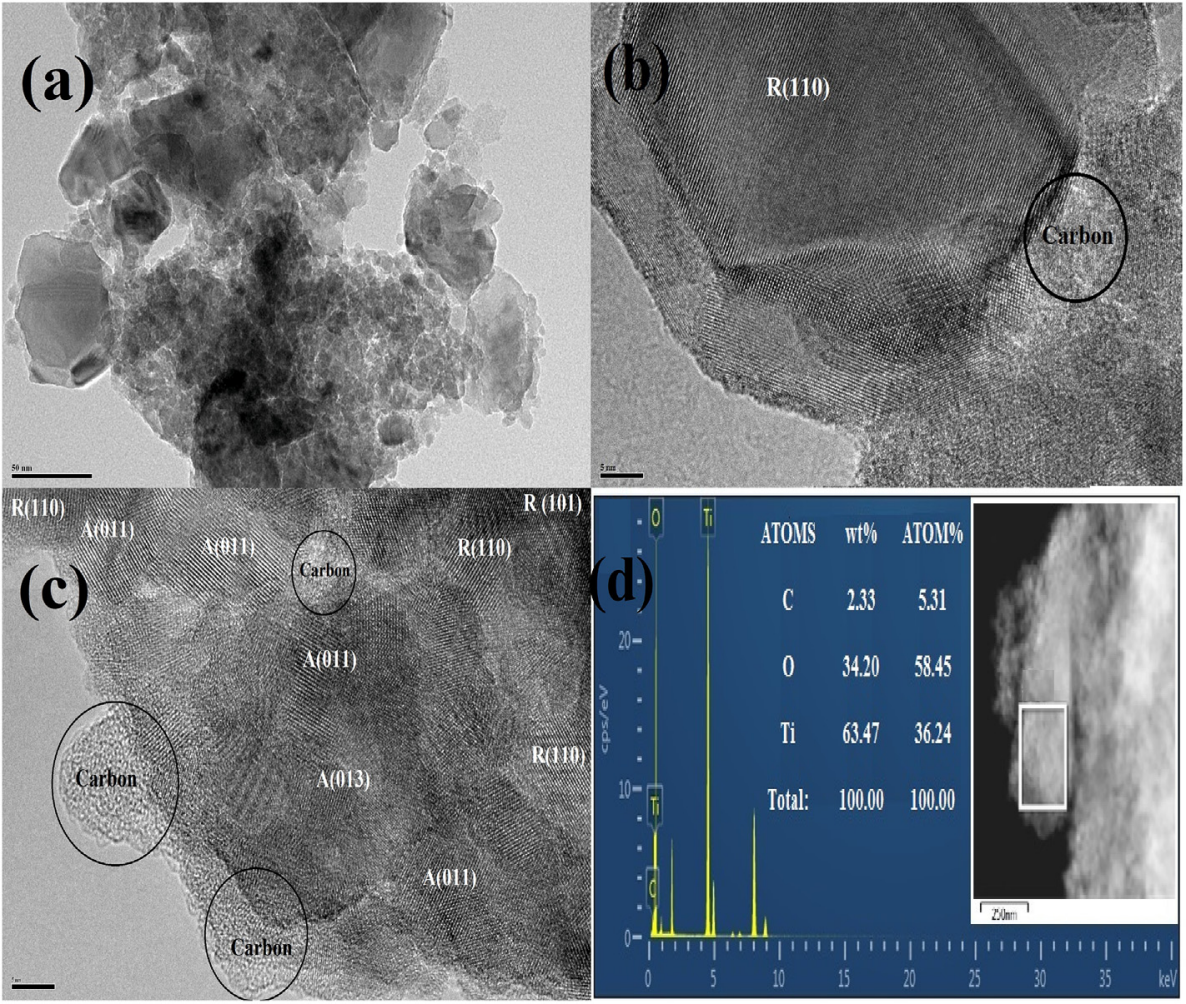
(a) HRTEM image for sample TD550, (b) Image of a large particle with rutile phase, (c) cluster of grains with anatase structure, and (d) chemical compositions with EDAX. The scale bar is 50 nm for (a) and 5 nm for (b and c).

The microstructures and morphology of titania nanopowder samples were characterized using HRTEM. Figure 5 shows the obtained microstructures for the sample TD550.

**Figure 6:**
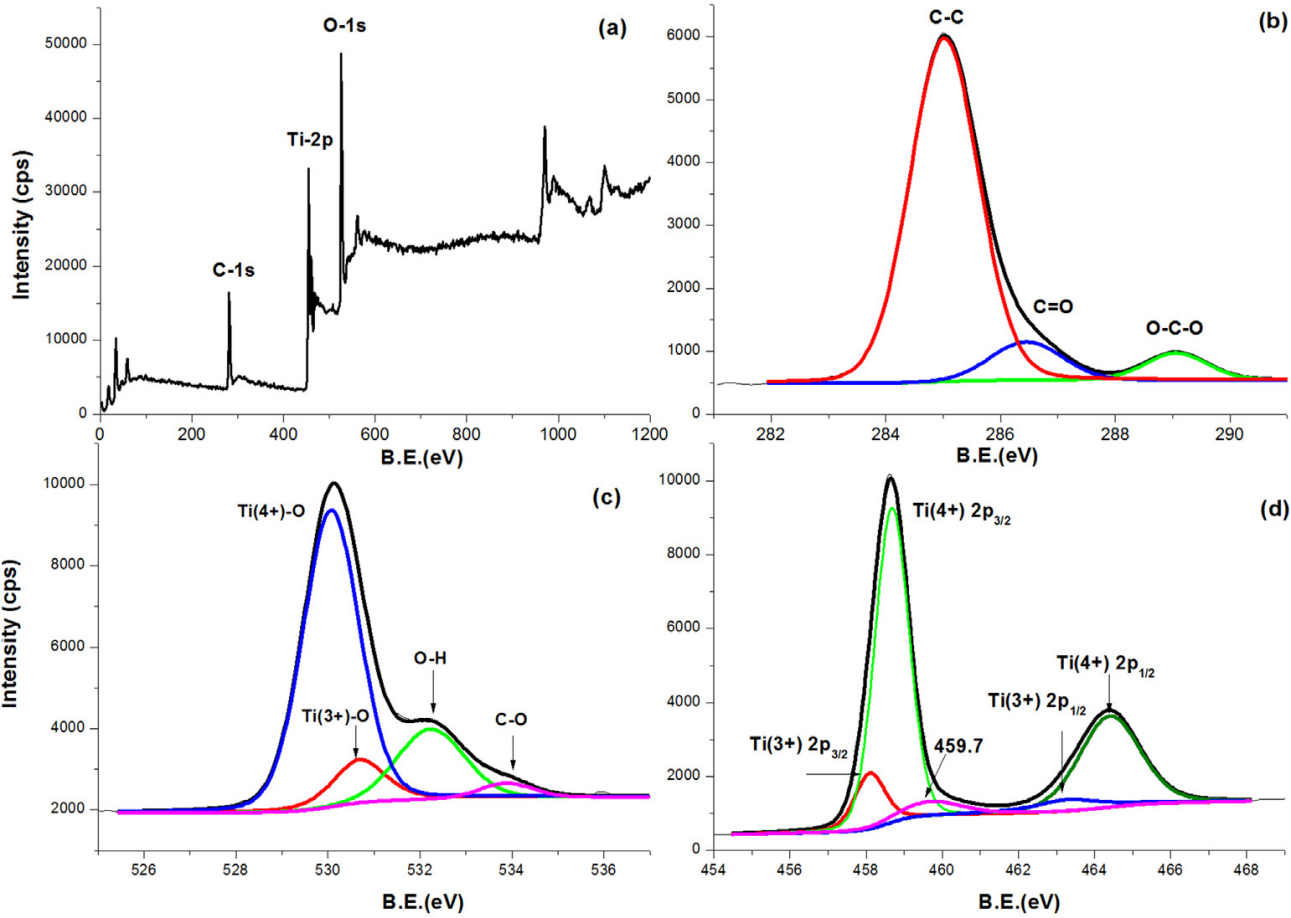
High-resolution XPS survey (a) C1s (b) O1s (c) Ti2p, and (d) photoelectron peaks of the sample TD550.

The particle size of the rutile phase and the large particles in Figures 5 a and b are comparable to those calculated using the W–H equation; however, the particle size of the anatase phase looks smaller. Figure 6(b) and (c) with scale bar nm show a higher resolution micrograph demonstrating rutile phase particles and anatase grain clusters cemented with amorphous carbon on the surface, respectively. Figure 6(d) shows the chemical compositions of the samples examined using EDAX attached to the HRTEM. The ratio O/Ti was <2, indicating that titania nanopowders are nonstoichiometric. This can be explained because the surface area subjected to the electron beam is extremely small, and the electron beam may encounter an area with a high concentration of oxygen vacancies or have TiO_2_ atomic plains with a lower O/Ti ratio. The carbon content is relatively larger than that estimated by TG measurement, which may be attributable to higher carbon density on the sample grid. It is thus subjected to an electron beam beside the carbon content.

### 3.5 X-ray photoelectron spectroscopy analysis

**Figure 7:**
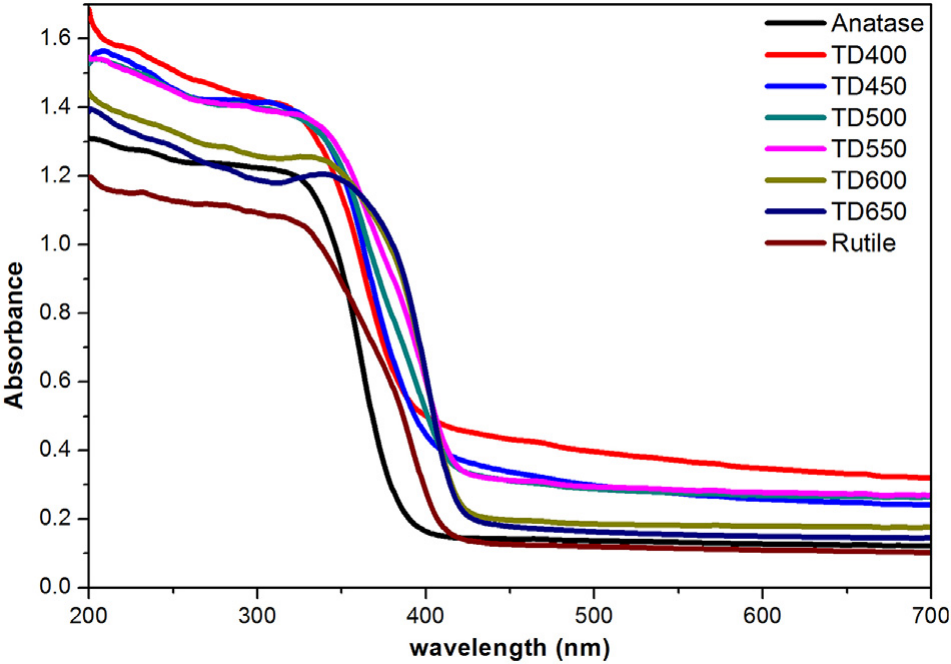
Absorbance spectra of certain selected samples and commercial anatase and rutile powders.

Figure 7(a) shows the XPS survey spectrum for the composite samples, revealing the characteristic photoelectron peaks C1s, O1s, and Ti 2p of carbon, oxygen, and titanium with binding energies of 284.8, 458.5, and 529.7 eV, respectively. The characteristic photoelectron peak of nitrogen N1s was not detected to indicate that nitrogen is not incorporated in the chemical composition.

**Figure 8:**
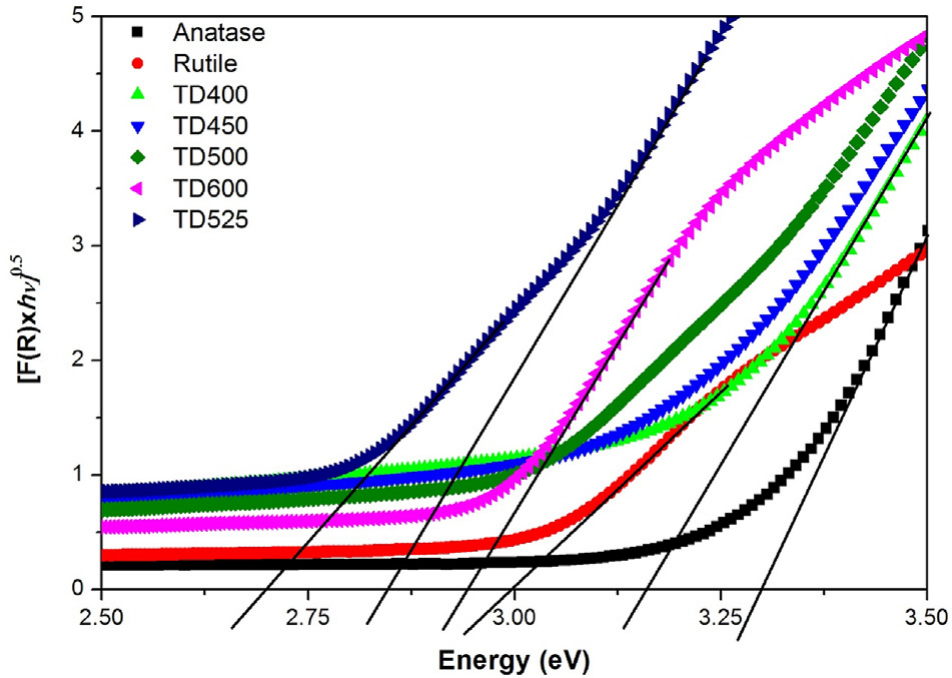
Bandgap energy calculations using the plot of the Kubelka–Munk function versus the photon energy of light absorbed by certain selected samples and commercial anatase and rutile.

XPS C1s are fitted with three peaks and shown in Figure 8(b); binding energy values of the main peak at 285.05 eV are attributed to the C–C bond with the sp^2^ orbital, revealing the presence of elemental carbon, while the other peaks at 286.5 and 289 eV corresponds to the C = O and O–C–O bonds, which reveals the presence of surface carbonates and/or structural fragments such as Ti–O–C and Ti–OCO [51, 52]. The binding energies at 281.9 and 290.7 eV corresponding to carbide (C–Ti bond) and graphitic carbon, respectively, are not detected in all samples, indicating that carbon is not incorporated as a dopant element in the TiO_2_ lattice and that there are no graphitic carbon structures formed on the surface of TiO_2_ particles, with amorphous carbon being the only pure carbon structure present [10, 14].

Figure 7 (c) shows the O 1s peak as fitted into four peaks centered at 530.07, 530.7, 532.23, and 533.9 eV. The lower energy peak at 530.07 eV is attributed to O 1s in the Ti–O linkages of TiO_2_. In comparison, the smaller peak at 530.7 is attributed to Ti^(3+)^–O linkage as that of Ti_2_O_3_, the peak at ∼532.23 eV is attributed to OH groups, and the weak peak at 533.9 eV is attributed to covalent oxygen resulting from incomplete oxidation of carbon sources [53, 54]. The Ti2p photoelectron spectrum is fitted with five peaks centered at 458.1, 458.7, 459.7, 463.3, and 464.4 eV, respectively, as shown in Figure 7(d). The peaks located at 458.1 and 463.3 eV are attributed to Ti3+ 2p_3/2_ and Ti3+ 2p_1/2,_ respectively; however, the peaks at 458.7 and 464.4 eV are typical of 2p_3/2_ and Ti 2p_1/2_. The difference between them was 5.7 eV, which corresponds to the normal state of Ti^4+^ in TiO_2_ [55]. Other researchers have identified a weak peak centered at 459.7 eV because of deconvolution and fitting. Sarkar *et al.* attributed the 459.7 eV peak on the Ti2p spectrum to the (Ti3+2p_1/2_), indicating the presence of oxygen vacancies and related defects on the surface of H_2_Ti_3_O_7_ nanowires [56], while Ekemena *et al.* suggested that this peak might be attributed to Ti–C bond on the surface [3, 57].

### 3.6 UV-vis spectroscopy

**Figure 9:**
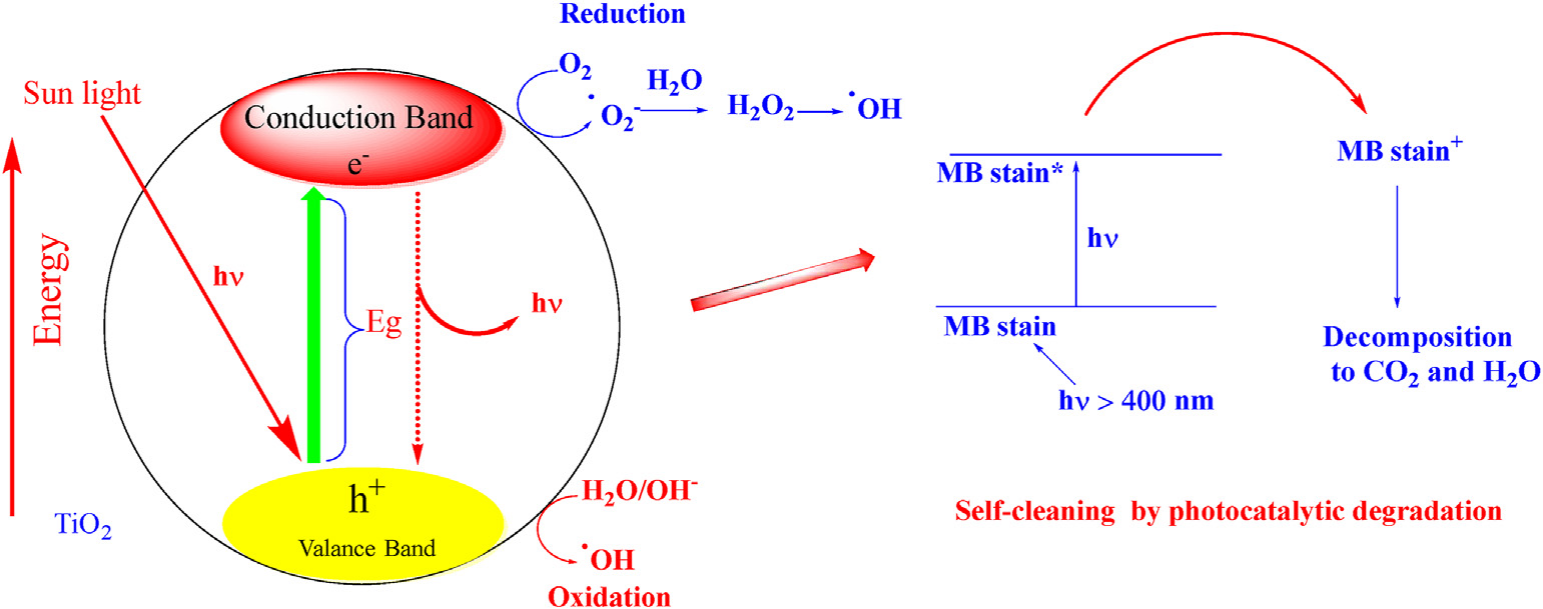
TiO_2_ nanocomposite photocatalytic degradation and photoexcitation to produce reactive oxidation species, and self-cleaning of MB stain.

UV-vis absorption and diffuse reflectance spectra (DRS) were conducted to evaluate the optical properties of prepared samples compared to the commercial pure anatase and rutile powders. Figure 9 shows the absorption spectra of carbon-containing titania samples calcined at different temperatures, anatase, and rutile powders.

All samples show absorption in both the UV and visible light regions higher than that of anatase and rutile. The sample TD400 heat-treated at 400 °C has the highest absorption, while the sample TD650 has the lowest. The sample TD700 shows the optical characteristics typical of commercial rutile. Generally, the absorption of light by C/TiO_2-δ_ decreases as the calcination temperature of the prepared samples increases. The absorption edge is shifted to higher wavelengths (redshift) towards the visible light region. The relatively higher absorption in the visible region is attributed to the presence of amorphous carbon structures [3, 58] where its fraction composition decreases with increase in calcination temperature.

The bandgap energies were calculated using the diffused reflectance (DR) data converted to the Kubelka–Munk function ${\left[F\left(R\right)h\upsilon \right]}^{n}$, where $F\left(R\right)=\frac{{\left(1-R\right)}^{2}}{2R}$ , n = 0.5 and 2 for direct and indirect bandgap transitions, respectively [59], from the intercept of the tangent to the curve with energy axes in the region where ${\left[F\left(R\right)h\upsilon \right]}^{n}\;$ linearly changes with photon energy(hυ). Figure 8 shows ${\left[F\left(R\right)h\upsilon \right]}^{n}\;$ as a function of absorbed photon energy by certain selected composite samples, pure anatase and rutile powders.

Table 2 contains the calculated bandgap energy values. The bandgap energies of commercial anatase and rutile are 3.3 and 3.0 eV, respectively. Bandgap energies are lower in samples heat treated at different temperatures from 400 °C to 650 °C than in reference materials. The lowest bandgap energy is recorded for the sample TD525 (42.54% A, 57.46% R), which is 2.695 and 2.829 eV for the rutile and anatase fractions, respectively. The change in bandgap energy is attributed to the change in fractional composition, oxygen vacancies, and trapped states of (Ti^3+^) [10, 38].

### 3.7 Self-cleaning action of fabrics loaded with titanate nanocomposite

Figure 9 illustrates how the photocatalytic destruction of titanium dioxide can be used to explain the self-cleaning behaviour of samples that have absorbed methylene blue (MB) stain [60, 61].

Table 3 displays the K/S values for the exposed and untouched regions of the samples throughout the course of 12–48 h. The self-cleaning capabilities of control samples and coated samples have been compared under the same testing conditions. The decrease in K/S percentage values of the exposed sample section compared to the unexposed sample portion was determined to evaluate the self-cleaning impact. Table 3 displays the K/S values for the exposed and untouched regions of the samples throughout the course of 12–48 h. The self-cleaning capabilities of control samples and coated samples have been compared under the same testing conditions. The decrease in K/S percentage values of the exposed sample section compared to the unexposed sample portion was determined to evaluate the self-cleaning impact.

**Table 3:** K/S decrease in methylene blue (MB) degradation as a percentage of sunshine exposure.

Fabrics sample	% Decrease in K/S value with exposure time					
	After 12 h		After 24 h		After 48 h	
	Untreated fabrics	Titanate nanocomposite treated fabrics	Untreated fabrics	Titanate nanocomposite treated fabrics	Untreated fabrics	Titanate nanocomposite treated fabrics
Cotton 100%	4.68	26.3425	4.09	34.27	3.88	39.36
Viscose	8.09	40.6625	6.08	46.25	4.18	50.755
Linen	8.16	51.6675	6.88	72.04	4.89	73.24
Cotton/PET	6.18	32.61	5.34	38.49	4.18	43.9

The MB stain fades quickly within the first 12 h of exposure, according to Table 3. The surface formation of reactive oxidation species is the basis for the self-cleaning ability of cellulose-based fabrics treated with TiO_2_NPs. When the sample is exposed to visible light, an excited methylene blue dye (MB) molecule adhering on the surface transfers an electron to the TiO_2_NPs' conduction band. As a result, the photocatalytic degradation of MB stain is caused by a reactive oxidation species produced by the electron caught by molecular oxygen on the surface of TiO_2_NPs.

**Figure:**
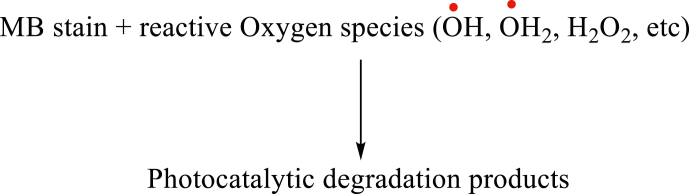

Here, the presence of TiO_2_ nanoparticles determines which titanate nanocomposite is responsible for the discoloration of "MB. This is explained by an increase in the band gap of irradiated TiO_2_ nanocomposite. A positive hole (h^+^) is left behind when the electron is stimulated from the valence band to the conduction band. The reduction and oxidation reactions are brought on by the electrons (e^−^) in the conduction band and the positive holes (h^+^) in the valence band, respectively (Figure 5). This causes the reactive oxygen O_2_ and OH species to develop, which can react with hazardous organic compounds and break them down into less harmful byproducts [62].

## 4 Conclusion

Nonstoichiometric carbon containing titania nanocomposites has been successfully synthesized via a facile, fast, and green sol–gel route. The sol–gel method allows the regulation of the crystallite size, the weight fraction of each crystalline phase, and carbon content. Diethanolamine (alkanolamine) is an essential in situ carbon source and its functions as a stabilizer for (Ti^4+^) cations and a pH modifier. The phase transformation from amorphous to rutile phase over a short range of temperatures occurs stepwise from amorphous to (anatase/brookite), to (anatase/rutile), and then to the pure rutile phase. The increase in calcination temperature indicates that the amorphous carbon content decreases and completely transforms into a pure rutile phase. Significant improvement in the electronic and optical absorption properties of titania nanocomposites compared to ordinary titania nanopowder was observed, making it more effective in many technological applications; this improvement is strongly correlated with structure, composition, defects, and impurities. The synthesized titanate nanocomposite sol-gel method shows higher photocatalytic activity (self-cleaning effect). Titanate nanocomposite-treated fabrics show promise as a potential application for both industrial and medical uses.

## Declarations

### Author contribution statement

Ibrahim Moussa, Hassan Ibrahim, El-Amir M. Emam, and Tawfik M. Tawfik: Conceived and designed the experiments; Performed the experiments; Analyzed and interpreted the data; Contributed reagents, materials, analysis tools or data; Wrote the paper.

### Funding statement

This work was supported by the National Research Centre (project number: E120302).

### Data availability statement

Data will be made available on request.

### Declaration of interests statement

The authors declare no conflict of interest.

### Additional information

No additional information is available for this paper.
